# Unexpected relationships of substructured populations in Chinese *Locusta migratoria*

**DOI:** 10.1186/1471-2148-9-144

**Published:** 2009-06-28

**Authors:** De-Xing Zhang, Lu-Na Yan, Ya-Jie Ji, Godfrey M Hewitt, Zu-Shi Huang

**Affiliations:** 1 State Key Laboratory of Integrated Management of Pest Insects and Rodents, Institute of Zoology, Chinese Academy of Sciences, Beijing 100101, PR China; 2 Center for Computational and Evolutionary Biology, Institute of Zoology, Chinese Academy of Sciences, Beijing 100101, PR China; 3 School of Biological Sciences, University of East Anglia, Norwich NR4 7TJ, UK; 4 Current address: College of Biological Science and Engineering, Hebei University of Science and Technology, Shijiazhuang, Hebei 050018, PR China

## Abstract

**Background:**

Highly migratory species are usually expected to have minimal population substructure because strong gene flow has the effect of homogenizing genetic variation over geographical populations, counteracting random drift, selection and mutation. The migratory locust *Locusta migratoria *belongs to a monotypic genus, and is an infamous pest insect with exceptional migratory ability – with dispersal documented over a thousand kilometers. Its distributional area is greater than that of any other locust or grasshopper, occurring in practically all the temperate and tropical regions of the eastern hemisphere. Consequently, minimal population substructuring is expected. However, in marked contrast to its high dispersal ability, three geographical subspecies have been distinguished in China, with more than nine being biologically and morphologically identified in the world. Such subspecies status has been under considerable debate.

**Results:**

By multilocus microsatellite genotyping analysis, we provide ample genetic evidence for strong population substructure in this highly migratory insect that conforms to geography. More importantly, our genetic data identified an unexpected cryptic subdivision and demonstrated a strong affiliation of the East China locusts to those in Northwest/Northern China. The migratory locusts in China formed three distinct groups, viz. (1) the Tibetan group, comprising locusts from Tibet and nearby West China high mountain regions; this is congruent with the previously recognized Tibetan subspecies, *L. m. tibetensis*; (2) the South China group, containing locusts from the Hainan islands; this corresponds to the Southeast Asia oriental tropical subspecies *L. m. manilensis*; (3) the North China group, including locusts from the Northwest and Northern China (the Asiatic subspecies *L. m. migratoria*), Central China and Eastern China regions. Therefore, the traditional concept on *Locusta *subspecies status established from Uvarov in 1930s needs to be revised. The three groups of locusts probably have separate evolutionary histories that were most likely linked to Quaternary glaciations events, and derived from different ancestral refugial populations following postglacial expansions.

**Conclusion:**

The migratory locust populations in China have differentiated into three genetically distinct groups despite high dispersal capability. While this clarified long-standing suspicions on the subspecific diversification of this species in China, it also revealed that the locusts in the vast area of East China are not the oriental subspecies but the Asiatic subspecies, an unexpected substructuring pattern. The distribution pattern of the three locust groups in China may be primarily defined by adaptive differentiation coupled to Quaternary glaciations events. Our results are of general significance both for locust research and for phylogeographical study of flora and fauna in China, illustrating the potential importance of phylogeographical history in shaping the divergence and distribution patterns of widespread species with strong dispersal ability.

## Background

Patterns of population genetic differentiation of an organism are shaped by various factors, such as geographical barriers, ecological difference, and historical processes, as well as the dispersal ability of the species. Highly migratory species are usually expected to have minimal population substructure over their distributional ranges because strong gene flow can counteract the isolating effects of geographical distance and physical barriers, and even remove genetic differentiation due to local adaptation [1]. However, historical processes such as climatic fluctuations and geological events can modify their range, leading to population subdivision even in species with high dispersal capabilities, as seen in some large mammals [2] and insects [3].

The migratory locust *Locusta migratoria*, belonging to a monospecific genus, is one of the most important agricultural pests in the world, and outbreaks were recorded as early as in 13^th ^century BC [4]. Its distributional area is greater than that of any other locust or grasshopper [5], occurring in practically all the temperate and tropical regions of the eastern hemisphere (Asia, Europe, Africa and Australasia) from 154 m below sea level in Xinjiang (Sinkiang) to about 4,600 m above sea level on the Tibetan plateau [6]. Such a vast distribution and monospecific status suggest exceptional migratory ability, and indeed dispersal over a thousand kilometers has been documented [7,8]. Consequently, minimal population substructure is expected within its distributional range. Nevertheless, some nine geographical subspecies have been distinguished biologically and morphologically [9-12], three of which are present in China, viz: *Locusta migratoria migratorioides*, *L. m. tibetensis*, and *L. m. manilensis*. There is considerable debate on the reliability of the subspecific status so identified [11,13,14], since morphological characters are readily influenced by regional climatic and habitat variation, and identification of subspecies has often been based on locality rather than critical examination of specimens [5]. This is particularly so for locusts in China with perennial doubts of their subspecific affinities [11,14].

Additionally, phylogeographical study in China as a whole is still underpresented [15], and genetic data are particularly scanty for deducing what has happened to the biomes during glaciations and deglaciations in past Quaternary cycles in China. This further impinges on the origins of species in neighboring areas in the Palaearctic and also North America [16,17], and is an important issue for understanding global effects of Pleistocene climate variations [18]. Thus, large geographical scale phylogeographical study in East Asia is pressingly needed and likely to reveal hidden patterns of biogeographic evolution. Here we present an extensive population genetic survey of the migratory locust in China using highly polymorphic microsatellite DNA markers. We aim to explore the following questions: (1) Whether the subspecific patterns morphologically identified for the migratory locust in China are genetically supported; (2) How to explain the patterns in the context of biogeographic evolution, given the strong dispersal capabilities of the insect? We provide robust genetic evidence for strong population structure and an unexpected cryptic subdivision in this insect in China, clarifying some long-standing issues in the subspecific divergence of this insect. We suggest that historical phylogeographical factors and the associated ecological adaptation have played important roles in shaping the observed genetic and geographic patterns in this highly migratory insect.

## Results

**Figure 1:**
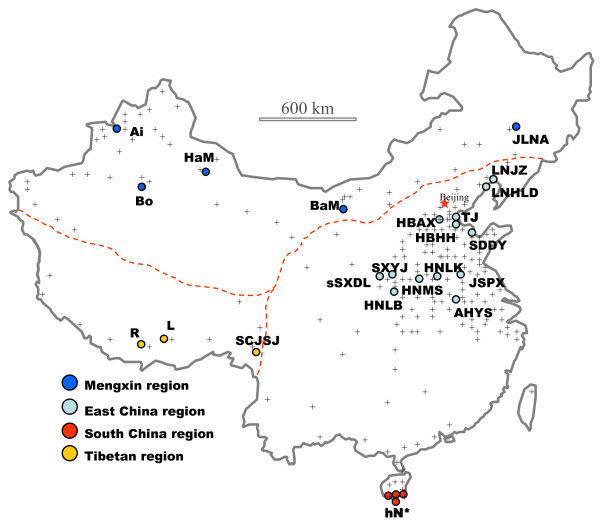
**Distribution and sample location of the migratory locusts in China**. 'Crosses (+)' in the background represent observed distribution sites of the migratory locusts. Color circles indicate localities of samples studied in this paper: Blue for samples from the Mengxin region, green for East China, red for South China, and orange for Tibetan region. hN* refers to the four samples from Hainan (hNLD, hNSYms, hNSY1 and hNSY2). Dashed line in the map schematically indicates the traditional view of the geographic boundary of the three subspecies in China. Please refer to Table 1 for the geographic origins of locust samples.

In total, 1381 individual locusts from 26 localities (Figure 1) were genotyped at eight nuclear microsatellite loci [19]. After corrections for multiple comparisons, no linkage disequilibrium was detected for the eight loci employed, but most population samples (23 out of 26) deviated from Hardy-Weinberg equilibrium (HWE) at one to five loci. Micro-Checker identified that the presence of null allele(s) potentially contributed to this deviation. Estimated frequencies of null alleles per locus per sample ranged from 0 to 0.438 (only in five cases, the value was >0.4), and in 112 of the 208 locus-sample combinations the values were greater than 0.05. The most frequent null allele frequencies were in the range of 0 – 0.1. The average null allele frequencies over loci (F_null_) vary from 0.048 (BaM) to 0.217 (F) among population samples (Table 1). At locus LmIOZc36, null alleles were detected in all 26 samples (with the frequency ranging between 0.208 – 0.429). If this locus was excluded, nearly half of samples were in HWE at the remaining seven loci. Three complementary approaches were performed to examine the relationships of the locust populations studied, i.e. genetic distance based neighbor-joining approach, Bayesian inference and principal component analysis. There was no significant change in results if the locus LmIOZc36 was excluded from the data. Each analysis was conducted separately on two data sets: the original data set without correction for null alleles and the data set corrected for null alleles. Since highly congruent results were obtained in such analyses, we only show the results from the original data set.

**Table 1:** Pair-wise F_ST_ values of locust populations studies here (upper triangle: + statistically significant at 0.05 level; lower triangle: pair-wise F_ST_; within-region comparisons are shown in bold print)

Population No. & code		Locality	Regional label	Sample size	MNA	H_E_	H_O_	F_null_
1	Ai	Aibi Lake, Xinjiang	Mengxin	29	16.75	0.825	0.649*	0.089
2	JLNA	Nong'an, Jilin	Mengxin	18	12.50	0.811	0.693	0.074
3	BaM	Bameng, Inner Mongolia	Mengxin	33	17.25	0.792	0.729	0.048
4	Bo	Bositeng Lake, Xinjiang	Mengxin	16	8.88	0.828	0.648*	0.100
5	HaM	Hami, Xinjiang	Mengxin	28	13.00	0.777	0.600*	0.101
6	HBAX	Anxin, Hebei	East China	55	19.75	0.823	0.621*	0.101
7	HBHH	Huanghua, Hebei	East China	113	24.88	0.814	0.654*	0.093
8	HNLB	Lingbao, Henan	East China	58	21.00	0.828	0.689*	0.086
9	HNLK	Lankao, Henan	East China	60	19.63	0.828	0.700*	0.072
10	HNMS	Mangshan, Henan	East China	35	18.13	0.768	0.665*	0.085
11	SDDY	Dongying, Shandong	East China	48	19.38	0.820	0.729	0.057
12	SXYJ	Yongji, Shanxi	East China	103	24.38	0.828	0.710*	0.070
13	sSXDL	Dali, Shannxi	East China	135	27.00	0.832	0.733*	0.060
14	TJ	Nandagang, Tianjin	East China	42	18.63	0.819	0.721*	0.059
15	AHYS	Yingshang, Anhui	East China	101	21.00	0.832	0.686*	0.082
16	JSPX	Peixian, Jiangsu	East China	78	22.25	0.795	0.691*	0.073
17	LNHLD	Huludao, Liaoning	East China	56	21.38	0.838	0.713*	0.069
18	LNJZ	Jinzhou, Liaoning	East China	88	22.75	0.837	0.680*	0.087
19	hNLD	Ledong, Hainan	South China	73	19.13	0.882	0.568*	0.166
20	hNSYms	Sanya, Hainan	South China	20	13.38	0.866	0.600*	0.145
21	hNSY1	Sanya, Hainan	South China	26	16.38	0.904	0.537*	0.165
22	hNSY2	Sanya, Hainan	South China	33	15.88	0.890	0.570*	0.185
23	L	Lasa, Tibet	Tibetan	38	10.75	0.739	0.573*	0.129
24	SCJSJ	Jinshajiang, Sichuan	Tibetan	13	7.13	0.780	0.541*	0.141
25	R	Rigeze,, Tibet	Tibetan	63	12.38	0.826	0.498*	0.178
26	F	Eritrea, Africa	Africa	19	12.00	0.898	0.466*	0.217

*Samples deviated from Hardy-Weinberg equilibrium. Locusts in 'Mengxin' and 'East China' regions are collectively referred to as 'North China' group.

**Figure 2:**
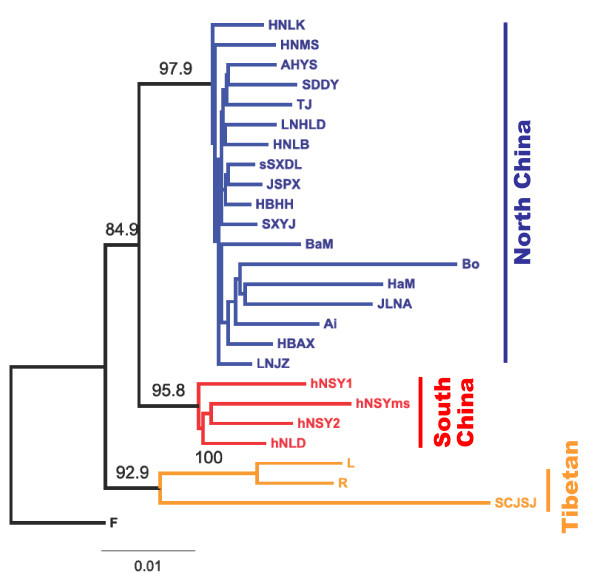
**Neighbor-joining tree illustrating the relationships of the migratory locust populations in China based on allelic frequencies at eight microsatellite loci**. This is a tree based on Cavalli-Sforza's chord distance (Dc) from the microsatellite loci. The tree based on Nei's standard genetic distance (Ds) has highly concordant topology and thus is not shown. Numbers above branches are percentage bootstrap support values from 1000 replicates (only values > 50% shown). The African population 'F' was used as the outgroup. Color codes: Blue for samples from North China (Mengxin region + East China), red for South China, and orange for Tibetan region.

Figure 2 shows the neighbor-joining population tree based on Cavalli-Sforza's chord distance (Dc) from the microsatellite loci [tree based Nei's standard genetic distance (Ds) has highly concordant topology]. It indicates that the Chinese locust populations studied are clustered genetically into three major groups each with strong bootstrap support: (1) the Tibetan group (orange clade in Figure 2), comprising locusts from Tibet and nearby West China high mountain regions (R, L and SCJSJ); this is congruent with the previously recognized Tibetan subspecies, *L. m. tibetensis *[12]; (2) the South China group (red clade), containing locusts from the Hainan islands (hNLD, hNSYms, hNSY1, and hNSY2); this corresponds to the Southeast Asia tropical subspecies *L. m. manilensis *[11]; (3) the North China group (blue clade), including locusts from the Northwest China (NWC), Northern China (NC), Central China (CC) and Eastern China (EC) regions, which are originally assigned as *L. m. migratoria *(NWC & NC) and *L. m. manilensis *(CC & EC).

Additional file 1 (Table S1) gives the pairwise F_ST _values between the locust populations. F_ST _values between populations of different groups range between 0.023 – 0.149, and are significantly different from zero, indicating that the three groups are moderately to strongly differentiated (Additional file 1 (Table S1)). Within each group, populations are genetically similar; F_ST _values among populations in general are small (0.000 – 0.010) and not significantly different from zero for most pair-wise comparisons (excepting the Tibetan group and some populations of the North China group, see below) (Additional file 1 (Table S1)).

**Figure 3:**
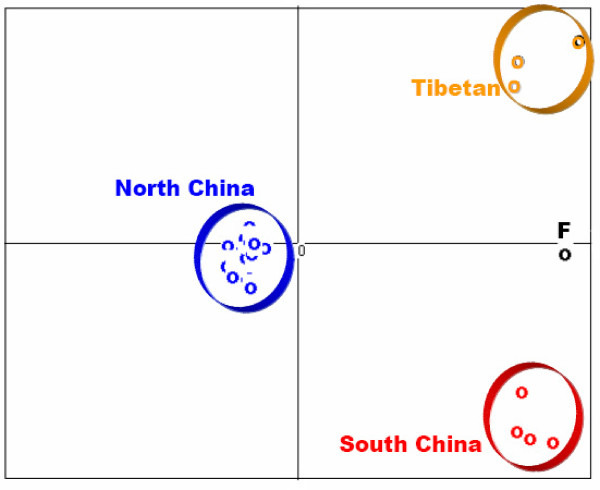
**Results of principal component analysis (PCA) generated from microsatellite data in PCA-GEN**. The first two principle component factors (PC1 and PC2) plotted here account for 63.7% of the total variance (per axis inertia: PC1 = 47.38, PC2 = 16.32). These two factors are highly significant based on statistical tests with 1000 randomizations. Colors are coded as in Figure 2. Note that the North China group symbols cannot be fully seem due to overlapping.

The results of principal component analysis (PCA) of the microsatellite genotype data are shown in Figure 3. The Chinese locust populations form three clusters "Tibetan", "South China" and "North China", which is identical to patterns seen in the neighbor-joining population tree based on Dc (Figure 3).

**Figure 4:**
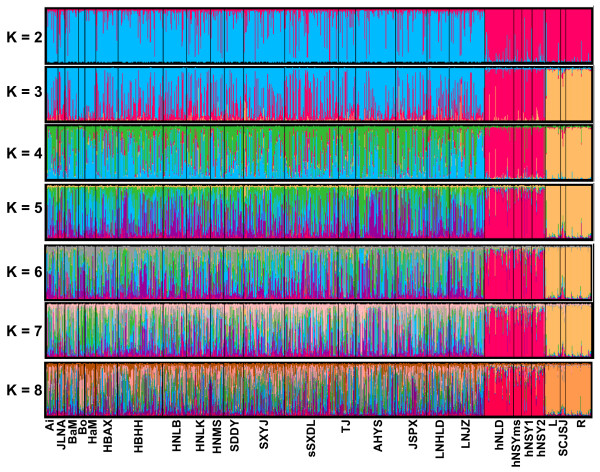
**Bayesian estimation of population structure**. *K *is the number of distinct clusters (groups) simulated with the data. Each of the 1362 locust individuals (African samples not included) is represented by a thin vertical line, which is partitioned into colored segments that represent the individual's estimated membership fractions in *K *clusters. Black lines separate different populations. Population labels are below the figure. Colors are coded as in Figure 2.

Figure 4 shows the results of Bayesian STRUCTURE analysis. It also inferred three clusters (*K *= 3) for the Chinese locust populations (the mean Dirichlet parameter Alpha (*α*) for degree of admixture is 0.041 at *K *= 3), corresponding to the three major groups identified in the aforementioned phylogenetic approach (Figure 2) and PCA analysis (Figure 3). At various defined *K *values (simulated from 2 to 8, Figure 4), the Tibetan group and the South China group each remains as a fixed cluster (except at *K *= 2, where these two groups merged as one cluster, with the rest of the populations as the other cluster). At higher *K *values (4–8), the locusts in North China group keep splitting further, albeit apparently irregularly (Figure 4).

Table 2 displays the results of analysis of molecular variance (AMOVA) of the microsatellite data. AMOVA was used here to examine which grouping of the Chinese locust has the maximum among group variance, and whether the traditional taxonomic classification of the Chinese locust has a high among-group variance. The maximum among-group variance (5.11%) was obtained only when populations are partitioned as Tibetan, South China and North China groups as seen above. If the North China group is further divided into two subgroups, i.e. the Mengxin subgroup and East China subgroup, then AMOVA results show that the among-group variance between Mengxin and East China subgroups (0.24%) is insignificant compared to the within-group-among-population variance (0.26%). Thus, such a division makes no biological sense. In marked contrast, the among-group variance between East China subgroup and the South China group (3.93%) is about 36-fold that of their within-group-among-population variance (0.11%).

**Table 2:** Analysis of Molecular Variance (AMOVA)

Grouping structure*	Number of groups	Number of populations	Variance components (%)		
			
			Within populations	Among populations within groups	Among groups
Overall	1	25	97.56	2.44	-
North China/South China/Tibetan	3	25	94.33	0.56	5.11
Mengxin/(East + South China)/Tibetan	3	25	95.48	1.38	3.14
East China/South China	2	17	95.96	0.11	3.93
North China/South China	2	22	95.77	0.28	3.95
(East + South China)/Mengxin	2	22	98.61	1.18	0.22
East China/Mengxin	2	18	99.5	0.26	0.24
South China/Mengxin	2	9	94.96	0.78	4.26
East China/Tibetan	2	16	94.53	0.43	5.04
Mengxin/Tibetan	2	8	90.24	3.16	6.06
South China/Tibetan	2	7	93.54	2.09	4.37

North China/Tibetan	2	21	92.69	0.59	6.72

* Groups are defined as follows: Overall = all population samples from China, North China = (East China + Mengxin), East China = (AHYS, HBHH, HBAX, HNLB, HNMS, HNLK, JSPX, LNHLD, LNJZ SDDY, SXYJ, sSXDL, TJ,), Mengxin = (Ai, BaM, Bo, HaM, JLNA), South China = (hNLD, hNSYms, hNSY1, HnSY2), and Tibetan = (L, R, SCJSJ). Traditionally, (East China + South China) has been considered to belong to the oriental subspecies. Please refer to Figure 1 for their location.

## Discussion

### Patterns of genetic differentiation and unexpected cryptic subdivision

The present multilocus microsatellite genotyping analysis studied 25 population samples from all three subspecies of the migratory locust in China, namely the Asiatic migratory locust *L. m. migratoria*, the oriental migratory locust *L. m. manilensis*, and the Tibetan migratory locust *L. m. tibetensis*. Overall, our data revealed that the migratory locust populations in China have differentiated into three distinct groups: the Tibetan clade (orange circles in Figure 1), the South China clade (red coded) and the North China clade (blue and green coded). This genetic pattern is concordant with geographic distribution, and was strongly supported by several complementary approaches (Figures 2, 3, 4 and Table 2, genetic distance-based phylogenetic approach, multivariate method, Bayesian clustering inference and variance analysis, respectively). We emphasize that the principal component analysis does not make strong assumptions of Hardy-Weinberg equilibrium, and Bayesian inference does not take into account the sample locations of individuals. The concordance between these approaches indicates the robustness of the patterns revealed. The above genetic pattern largely confirms the subspecific diversification in this species recognized from biological and morphological data [5,6,10,12]. A major, unexpected disagreement exists, however, between our genetic data and the traditional treatment of subspecific status of the locusts in East China (green circles in Figure 1). Traditionally, locusts in the immense area of East China and South China (e.g. Hainan islands, red circles in Figure 1) have been classified as the subspecies *L. m. manilensis *[5,6,10], this being an accepted concept since Uvarov's work in 1930s [10]. Our genetic data have identified a cryptic subdivision between these locusts, and demonstrate a strong affiliation of the locusts in East China to those in Northwest/Northern China (blue circles in Figure 1) instead of those in South China.

An issue related to the sampling scheme deserves some consideration before we can draw any firm conclusion from the above observations. In our study, all population samples from South China are from the Hainan islands with no sample from the adjacent continent. (Although the migratory locusts were recorded in continental South China, in most of the time they form only solitary populations of low density. We failed to obtain any sample from there after several attempts). Thus one possibility is that the observed population structure might reflect the effect of gene flow barrier between the island and the continent populations, with simply an artifact of insufficient sampling in South China. Several lines of evidence argue against this suggestion. First, the minimum distance between Hainan islands and the main continent is only 20 km (the width of the Qiongzhou Strait that separates the island and the continent varies between 20 to 30 km [20]), which does not form an effective barrier of gene flow. Long-distance migration of locusts in Hainan has been well documented [14]. Second, revisiting the earlier literature carefully revealed that in the 1990s ecologists and taxonomists had already noticed some subtle morphological differences between locusts in East China and South China (Hainan region) [14] and the somewhat closer affiliation in certain morphometric measures of the East China locusts to those in the Mengxin region [21]. For example, Ding questioned in 1995 whether the migratory locusts in East China are really the oriental subspecies as seen in Hainan, since the black strip marking on both sides of the pronotum found in the solitary locusts from Hainan were not present in the majority of locusts from East China [14] (however his view has received little attention). This lends independent support to our genetic findings. Third, there exists significant physiological difference in cold hardiness between North China populations and the Hainan populations, with the North China locusts being adapted to cold winter weather and the South China locusts to hot tropical climate [22].

Therefore, our genetic data confirmed earlier suspicions on the subspecies status of the migratory locusts in East China, and suggest that the traditional concept established from Uvarov [10] needs to be revised: the locusts in the vast area of East China are not the oriental subspecies but the Asiatic subspecies, and thus have a different evolutionary history from locusts in South China. Recently, Chapuis *et al*. [23] have reported the existence of intraspecific subdivision in this highly migratory insect by microsatellite DNA analysis of rangewide samples, which appeared to not correspond well to traditional subspecies taxonomy. Our results readily clarify some of the oddities observed in their study – viz. why do the oriental migratory locusts in East China (their collecting site no. 15) not cluster with their consubspecifics in Southeast Asia? This is because they belong to different subspecies.

### Phylogeographical implications of the observed differentiation patterns

Highly migratory species are usually expected to have minimal population substructure over their distributional ranges [24] because strong gene flow has the effect of homogenizing genetic variation over geographical populations, counteracting random drift, selection and mutation [1,24-26]. In contrast, both the traditional morphometric and our complementary genetic analyses demonstrated a largely concordant differentiation pattern of locust populations in China. This suggests that either the dispersal ability of the migratory locust is not as strong as thought (such that gene flow cannot effectively prevent geographical populations from drifting apart genetically), or some other processes are involved, which caused population divergence. However, the strong migratory ability of the migratory locust (especially long-distance migration) has been well documented [7,8]. Our results also revealed that populations separated over 1000 km in East China do not show genetic differentiation (Additional file 1) and there is no isolation by distance (IBD) within this region (data not shown). From classical population genetic theory, this indicates strong gene flow across large geographical area homogenizing populations, confirming the migratory locust as a strong disperser. It further indicates that geographical distance does not constitute a barrier for gene flow in this insect in China. Similarly, no physical barriers preventing locust migration seem to exist in East and South China, for example, locusts in East China and the Mengxin region are well connected despite the Taihangshan mountain chains (at 3,058 m) separating them.

Among the other processes likely involved in the divergence of the locust populations (*e.g*. habitat patchness, local extinction/recolonization events, phenological isolation, behavioral difference), we believe that historical process, such as historical climatic fluctuations played a primary role. The impact of Pleistocene glaciations cycles on floral and faunal distributions is now well recognized, being a major force shaping population diverging patterns in many organisms [16,17]. As a common scenario, populations were isolated in different refugial areas during glacial periods and diverged genetically from each others, subsequently extending their ranges by (re)colonization as the favourable climatic and ecological conditions resumed. This is also plausible for the migratory locust [27]. For example, in China in the mid-latitudes (30 – 40°N), at the last glacial maximum (LGM, ~20 kya), significant southward and eastward extension of steppe and desert biomes occurred. Cool mixed forests shifted *c*. 1,000 km eastward into the lowlands, and the northern boundary of broadleaved evergreen/warm mixed forests was displaced southward by *c*. 1,000 km [28]. Over the whole of north and east China, climatic conditions were much drier and colder in the LGM than today. A reduction of temperature between 7 to 12°C has been estimated [29,30], with a fall of sea level along the East China Sea coast up to 140 m [28]. Consequently, during the LGM in the areas where the North China group of locusts is found today, north to the latitude 38–40°N (eastern part) and 37–39°N (western part) were permafrost [31], and in the vast East China steppe and desert were the dominant vegetation types, with herbaceous plants being composed mainly of *Artemisia *and Chenopodiaceae [29,31-33]. These plants, which are indicators of cold conditions and also cause high death rate of hoppers (95%) or abortion of the moulting process [6,11,34], are not suitable food for the migratory locusts. Therefore, we can deduce that at the LGM the migratory locusts were very unlikely to survive in these areas. This means that locusts found today in these areas originated by recolonization from elsewhere after the LGM.

The most likely source of origin of locusts in North China is glacial refugia in the Black, Caspian and possibly Aral Seas basins regions, given that (1) recent climatic modelling studies have identified these regions as potential refugia areas along with the well known Iberian Peninsula, Italy, and the Balkans [35], (2) these regions have been shown to be refugia for many fresh water species [17,36], (3) the migratory locusts in these areas and North China belong to the same subspecies, *L. m. migratoria *[9,10,27], and (4) there existed independent evidence in scorpions that eastward postglacial expansion from the above regions to China was an important biogeographic component [37] (unpublished data, Shi CM & Zhang DX). Our proposition on the refugial area is reinforced by Chapuis *et al.'*s [23] recent study on rangewide *Locusta *populations from 25 collecting sites (including two sites in China, both in the northern region); they demonstrated a closer genetic affinity of North China locusts to those in central Asia. The lowland shores and surrounding reed-beds and deltas of several important rivers in these areas could serve as favorable breeding areas of the locusts during glacial times.

There is not enough evidence to deduce how the South China group of locusts (red circles in Figure 1) was affected by past glaciations. These locusts are most likely of Southeast Asia origin (locusts in Southeast Asia such as the nearby Indo-China Peninsula and the Philippines are all known as the tropical subspecies *L. m. manilensis*), considering recorded invasions of locusts from the Philippines to the Taiwan islands [10] and comparable tropical Savannah breeding habitat in these regions [14]. By contrast, the Tibetan group probably developed from local refugial sources. This group of locusts has a closer affinity to the outgroup, the African migratory locust, than the other two groups, and shows a strong within-group divergence pattern (Figures 2 and 3). Wright's F-statistics also indicate significant population differentiation within this group (Additional file 1; F_ST_>0.11 between SCJSJ and L/R), suggesting local isolation of geographical populations over a sufficiently long period of time. Pollen evidence suggests that the southern and eastern edges of the Tibetan plateau had favorable climatic conditions during the last glacial [38], being important refugial places for plants and animals. Thus, the present populations have probably been derived from glacial refugia in these areas, and local geography (high mountainous landscape) should have further enhanced genetic differentiation among populations. Interestingly, the present distribution pattern of the Tibetan group locusts largely parallels the distribution pattern of the broadleaved forests at LGM in Tibet, albeit shifted somewhat internally, and this is indicative of the refugial areas of the locusts and directions of postglacial expansion.

Therefore, circumstantial evidence suggests that the three genetically distinct locust groups in China were isolated from each other during evolution most likely coupled to Quaternary glaciation events, and were derived from different glacial refugial populations following postglacial expansions. Although we have focused our discussion above on LGM as this is the glaciation best understood, the differentiation patterns observed in the locust could well be a combined consequence of several glaciations cycles. Glacier studies in Tibetan Plateau have identified three major glaciation events in China in the Quaternary that were of great amplitude and left recognizable footprints (glacier relics) [39], including the LGM. Further study with DNA sequence data is clearly needed to more precisely estimate the time scale of differentiation of locust populations.

### Factors maintaining the current isolation of locust populations

How is the substructuring pattern of locust populations in China maintained given the strong dispersal ability of this insect? Distributional patterns of species are molded by a number of factors, including barriers to dispersal, physical and biological factors that make particular regions of habitat unsuitable for viability and/or reproduction [40]. The actual geographic distribution is defined by the complex interaction of the environment, the species fundamental ecological niche, and particular biological realities and historical events [41-46]. It is known that once populations have become genetically differentiated, their divergence status can be maintained if they have differentially adapted to regional ecological conditions, since geographic variation in selection can act as a strong barrier to gene flow [26,47]. This is likely the case to the migratory locust even though it is a strong disperser. The significant physiological difference in cold hardiness between North China (Mengxin region + East China) populations and the Hainan populations [22] reflects differential selection in this species in different regions potentially linked to historical isolation (see above). That is, the migratory locust populations in different refugial areas during glaciations periods could have undergone allopatric (or parapatric) divergence with adaptive evolution, and shifted to different adaptive landscapes. Thus, populations in Tibet have adapted to the ecological and climatic conditions at high altitude, the South China populations to subtropical and tropical conditions, and the North China populations to temperate conditions. This should have ecologically restricted their distributional ranges in postglacial expansions, and then prevented effective migration among ecologically different regions. Therefore, the current pattern of distribution of the three locust groups in China appears to be primarily defined by adaptive difference which has acted as barriers to gene flow. As a consequence, the current effective gene flow is weak and has little genetic consequence; that is, it is not strong enough to wipe out the patterns of differentiation created during historical isolation.

## Conclusion

In summary, the migratory locust populations in China have differentiated into three distinct groups despite high dispersal capability, and the locusts in the vast area of East China are not the oriental subspecies but the Asiatic subspecies. It suggests that these groups of locusts have separate evolutionary histories most likely molded by Quaternary glaciations events, and derived from different ancestral refugial populations following postglacial expansions. The population substructuring patterns observed in the migratory locusts, as reported here and in Chapuis *et al*. [23], are of general significance both for locust research and for phylogeographical study of flora and fauna in China and beyond, and are illustrating for widespread species with strong dispersal ability. In view of our sampling density and results obtained, it suggests that far more population samples are needed in order to study the worldwide population genetic structure and biogeographic evolution of highly mobile species, such as the migratory locust.

## Methods

### Sample collection and microsatellite genotyping

Locust samples studied here includes four subspecies based on locality and morphology, viz. the Asiatic migratory locust *L. m. migratoria *(occurring in temperate E. Europe to N. China, Korea and Japan), the oriental migratory locust *L. m. manilensis *(in E. and S. Asia and the Pacific region), the Tibetan migratory locust *L. m. tibetensis *(in Tibet and nearby W. China), and the African subspecies *L. m. migratorioides *(in Africa south of the Sahara and off shore Atlantic islands). The last one was used as the outgroup. Solitary *Locusta migratoria *individuals were collected between 1998 and 2001, stored either in DMSO-salt solution or in absolute ethanol at 4°C (Table 1). The Chinese insects were sampled over their distributional range across China (Table 1, Figure 1). In total, 1381 individual locusts from 26 localities were used in this study. Genomic DNA was extracted using a modified phenol-chloroform procedure as described by Zhang and Hewitt [48]. Each individual was genotyped at eight microsatellite loci [19] on an ABI PRISM™ 3100 Genetic Analyzer using Pop4 gel matrix with GENESCAN^® ^400HD (ROX) as the internal size standard. Sizes of the amplified microsatellites were scored by GeneScan 3.7 and manually checked for every allele. A blank control was carried out along each set of DNA extractions and PCR amplifications to monitor any possible cross contamination. Samples that did not amplify at more than two loci were excluded from further analysis.

Note that mitochondrial DNA of locusts is of little use for population genetic studies due to the presence of numerous pseudogenes in the nuclear genome [49], and nuclear ribosomal ITS regions do not contain enough sequence variation (DXZ's unpublished data).

### Data analysis

Heterogeneity testing was carried out for the two sexes and multiple samples collected from the same areas before pooling them in analysis, and no genetic difference was observed [50]. Basic population genetic parameters (the number of alleles, the observed and expected heterozygosity per locus) were estimated with MSTools 3.0 [51]. Hardy-Weinberg equilibrium (HWE) and linkage disequilibrium were tested using GENEPOP ver. 3.4 [52], GDA [53] and ARLEQUIN ver. 3.0 [54], with sequential Bonferroni correction for critical significance levels. Null alleles were examined with Micro-Checker [55].

Wright's F-statistics (F_ST _or *θ*), measures of population subdivision, were calculated using FSTAT 2.9.3 [56] and ARLEQUIN. Statistical significance of the estimates was evaluated by permutation or bootstrap procedure. Exact test of population differentiation has been carried out using GENEPOP, and the significance levels were assessed by Markov chain procedure.

AMOVA (Analysis of Molecular Variance) was performed using the program implemented in ARLEQUIN. AMOVA was used to examine which grouping of the Chinese locusts has the maximum among group variance, and whether the traditional taxonomic classification of the Chinese locust has a high among group variance. The traditional taxonomic classification is as follows (Figure 1; Table 1): Locusts in the Qinhai-Tibet plateau region belong to the Tibetan subspecies (*L. m. tibetensis*), locusts in Mengxin region in North China (Xinjiang, Inner Mongolia and Northeast China) the Asiatic subspecies (*L. m. migratoria*) and locust in East and South China the oriental subspecies (*L. m. manilensis*) [6,10,12]. This is equivalent to the grouping [Mengxin/(East China + South China)/Tibetan] (Table 2). The genetic analysis suggested the following grouping: [North China (= East China + Mengxin)/South China/Tibetan]. In addition to these two groupings, various other alternative groupings were also examined (Table 2), including: [East China/South China], [North China/South China], [(East + South China)/Mengxin], [East China/Mengxin], [South China/Mengxin], [East China/Tibetan], [Mengxin/Tibetan], [South China/Tibetan], [North China/Tibetan] and other multiple population combinations.

PHYLIP ver. 3.6 [57] was used for calculating the genetic distances and constructing population phylogenetic trees. Nei's standard genetic distance (Ds) and Cavalli-Sforza's chord distance (Dc) were estimated using the program GENDIST. Dc distance based tree topology is generally more robust for gene frequency data [58] and insensitive to null alleles [59]. 1,000 bootstrap replicates were performed to obtain statistical support for inferred trees.

A Bayesian clustering analysis implemented in the program STRUCTURE [60] was also used to infer population structure in the locust. This method allows the assignment of individual insects to distinct clusters based on their genotypes, without using sampling locations, hypothesized genetic origins of individuals or phenotypic information. Trial runs were first tested with varying length of iterations (10^4^–10^6^) after a burn-in period of various lengths (10^4^–10^6^). We found that stationarity was reached with a burn-in period of 1 × 10^4 ^iterations, and data collection for ≥1 × 10^5 ^iterations produced highly consistent results. Independent runs with different K values each with several replicates were then performed using a burn-in period of 1 × 10^5 ^iterations and data collection for 1 × 10^6 ^iterations, with a model of correlated allele frequencies. A criterion recommended for selecting the appropriate K value is the estimated posterior probability of the data, *P*(K/X) (see the program manual). For complex datasets with many groups, this criterion is difficult to apply. We have observed that the Dirichlet parameter *Alpha *(α) for degree of admixture appears to be a more reliable indicator of the 'correct' K value. For the clustering pattern with the most appropriate population structure (at the simulated 'correct K'), admixture among populations (the inferred clusters) should be minimal, and therefore α should be minimal; for values smaller or larger than the 'correct' K, α should always be larger. Thus, the smallest K with the smallest α is most likely the real structure contained in the data. It is expected that departures of data from HWE may lead to overestimating K. While this could particularly be a problem for closely related populations, it should have little influence on divergent populations. Graphical display of the results of STRUCTURE was done with the program DISTRUCT by N. A. Rosenberg [61].

Principal component analysis (PCA) was performed with PCA-GEN [62], incorporating 1,000 randomizations, and verified independently using the statistic software package SPSS ver. 10.0 (SPSS Inc., Chicago, IL, USA). As a complementary approach to model-based genetic analyses described above, this multivariate method does not make strong assumptions of Hardy-Weinberg equilibrium or linkage equilibrium in the data.

## Authors' contributions

LNY carried out the molecular genetic studies and participated in data analysis. YJJ participated in some field work, genotyping data collection, analysis and coordination, and helped to revise the manuscript. ZSH participated in some field work and genotyping studies. GMH coordinated the study and the Royal Society UK-China joint project, and helped to revise the manuscript. DXZ conceived of and designed the study, carried out field work, participated in data analysis and coordination, and wrote the manuscript. All authors read and approved the final manuscript.

## Supplementary Material

Additional file 1 **Table S1**. Pair-wise *F*_*ST *_values of locust populations studies here (upper triangle: + statistically significant at 0.05 level; lower triangle: pair-wise *F*_ST_; pop, population samples that are numbered as in Table 1; Neg, negative value, between -0.001 and -0.006) Click here for file

## References

[B1] Mayr E (1963). Animal Species and Evolution.

[B2] Brown DM, Brenneman RA, Koepfli KP, Pollinger JP, Mila B, Georgiadis NJ, Louis EE, Grether GF, Jacobs DK, Wayne RK (2007). Extensive population genetic structure in the giraffe. BMC Biol.

[B3] Schmitt T, Hewitt GM, Müller P (2006). Disjunct distributions during glacial and interglacial periods in mountain butterflies: *Erebia epiphron *as an example. J Evol Biol.

[B4] Fan YZ (1983). Locust outbreaks in the Shang Dynasty. Agri Archaeol.

[B5] Centre for Overseas Pest Research (1982). The Locust and Grasshopper Agricultural Manual.

[B6] Guo F, Chen YL, Lu BL (1991). The Biology of the Migratory Locusts in China.

[B7] Waloff ZV (1940). The distributions and migrations of *Locusta *in Europe. Bull Entomol Res.

[B8] Uvarov B (1977). Grasshoppers and Locusts.

[B9] Uvarov BP (1921). A revision of the genus *Locusta*, L. (= *Pachytylus*, Fieb.), with a new theory as to the periodicity and migrations of locusts. Bull Entomol Res.

[B10] Uvarov BP (1936). The oriental migratory locust (*Locusta migratoria manilensis*, Meyen 1835). Bull Entomol Res.

[B11] Uvarov B (1966). Grasshoppers and Locusts.

[B12] Chen YL (1963). A new subspecies of *Locusta migratoria *– Tibetan migratory locust (*Locusta migratoria tibetensis *subsp. n.). Acta Entomol Sin.

[B13] Farrow RA, Colless DH (1980). Analysis of the interrelationships of geographical races of *Locusta migratoria *(Linnaeus) (Orthoptera: Acrididae) by numerical taxonomy, with special reference to subspeciation in the tropics and affinities of the Australian race. Acrida.

[B14] Ding YQ, Liu JP (1995). Studies on the tropical Savannah locust breeding area in Hainan Island, a new oriental migratory locust breeding area in China. Studies on Acridoids of Hainan Island.

[B15] Beheregaray LB (2008). Twenty years of phylogeography – the state of the field and the challenges for the Southern Hemisphere. Mol Ecol.

[B16] Hewitt GM (2000). The genetic legacy of the Quaternary ice ages. Nature.

[B17] Hewitt GM (2004). Genetic consequences of climatic oscillations in the Quaternary. Phil Trans R Soc Lond B.

[B18] Kuchta SR, Meyer D (2001). A genealogical view of geographical variation. Mol Ecol.

[B19] Zhang DX, Yan LN, Ji YJ, Kang L, Hewitt GM, Huang ZS (2003). Isolation, characterization and cross-species amplification of eight microsatellite DNA loci in the migratory locust (*Locusta migratoria*). Mol Ecol Notes.

[B20] Li HC, Liu JP, Liu JP (1995). Physical environment of Hainan Island. Studies on Acridoids of Hainan Island.

[B21] Kang L, Chen YL (1991). The analysis of numerical taxonomy to the inter-relationships among different geographical populations of *Locusta migrtoria *phase solitaria (Orthoptera, Acrididae). Sinozoologia.

[B22] Jing XH, Kang L (2003). Geographical variation in egg cold hardiness: a study on the adaptation strategies of the migratory locust *Locusta migratoria *L. Ecol Entomol.

[B23] Chapuis MP, Lecoq M, Michalakis Y, Loiseau AG, Sword A, Piry S, Estoup A (2008). Do outbreaks affect genetic population structure? A worldwide survey in *Locusta migratoria*, a pest plagued by microsatellite null alleles. Mol Ecol.

[B24] Peterson MA, Denno RF (1998). The influence of dispersal and diet breadth on patterns of genetic isolation by distance in phytophagous insects. Am Nat.

[B25] Wright S (1931). Evolution in Mendelian populations. Genetics.

[B26] Slatkin M (1987). Gene flow and the geographic structure of natural populations. Science.

[B27] Zhang DX, Yan LN, Kang L, Ji YJ (2003). Some unorthodox views on the classification and evolution of the migratory locusts in China prompted by molecular population genetic study. Acta Zool Sin.

[B28] Yu G, Chen X, Ni J, Cheddadi R, Guiot J, Han H, Harrison SP, Huang C, Ke M, Kong Z, Li S, Li W, Liew P, Liu G, Liu J, Liu Q, Liu KB, Prentice IC, Qui W, Ren G, Song C, Sugita S, Sun X, Tang L, VanCampo E, Xia Y, Xu Q, Yan S, Yang X, Zhao J, Zheng Z (2000). Palaeovegetation of China: a pollen data-based synthesis for the mid-Holocene and last glacial maximum. J Biogeogr.

[B29] Tong GB, Zhang JP, Yan FH, Mai XS (1991). Sporeo-pollen sequence and division of climatic period in the eastern North China plain since late Pleistocene. Seismol Geol.

[B30] You YZ, Li KR (1992). Paleoclimatic change reflected in fauna and human culture. Climate Change and its Impact in China.

[B31] Cui ZJ, Yang JQ, Zhang W, Zhao L, Xie YY (2004). Discovery of a large area of ice-wedge networks in Ordos: Implications for the southern boundary of permafrost in the north of China as well as for the environment in the latest 20 kaBP. Chin Sci Bul.

[B32] Yang XD, Wang SM, Xue B, Tong GB (1995). Vegetational development and environmental changes in Hulun Lake since late Pleistocene. Acta Palaeontol Sin.

[B33] Zhang JH, Kong ZC, Du NQ (1999). Vegetation and environmrntal changes in the Fangshan area of Beijing from 16 000 – 7 000 years B.P. Acta Micropalaeontol Sin.

[B34] Kozhanchikov IV (1950). Fundamental features of food specialization in the Asiatic locust. Izv Akad Nauk USSR (Biol).

[B35] Leroy SAG, Arpe K (2007). Glacial refugia for summer-green trees in Europe and south-west Asia as proposed by ECHAM3 time-slice atmospheric model simulations. J Biogeogr.

[B36] Culling MA, Janko K, Boron A, Vasil'Ev VP, Cote IM, Hewitt GM (2006). European colonization by the spined loach (*Cobitis taenia*) from Ponto-Caspian refugia based on mitochondrial DNA variation. Mol Ecol.

[B37] Shi CM (2007). Phylogenetic and biogeographic evolution of the Chinese scorpion *Mesobuthus martensii *(Karsch 1879): A preliminary genetic study. PhD thesis.

[B38] Tang LY, Shen CM (1996). Late Cenozoic vegetational history and climatic characteristics of Qinghai-Xizang plateau. Acta Micropalaeontol Sin.

[B39] Shi YF (2000). Glaciers and Their Environments in China – the Present, Past and Future.

[B40] Burton R (1998). Intraspecies phylogeography across the Point Conception biogeographic boundary. Evolution.

[B41] Brown JH, Stevens GC, Kaufman DM (1996). The geographic range: size, shape, boundaries, and internal structure. Annu Rev Ecol Syst.

[B42] Patterson BD (1999). Contingency and detrminism in mammalian biogeography: the role of history. J Mammal.

[B43] Peterson AT, Soberón J, Sánchez-Cordero V (1999). Conservatism of ecological niches in evolutionary time. Science.

[B44] Anderson RP, Gómez-Laverde MP, Peterson AT (2002). Geographical distributions of spiny pocket mice in South America: insights from predictive models. Global Ecol Biogeogr.

[B45] Anderson RP, Martínez-Meyer E (2004). Modeling species' geographic distributions for preliminary conservation assessments: an implementation with the spiny pocket mice (*Herteromys*) of Ecuador. Biol Conserv.

[B46] Anderson RP, Peterson AT, Gómez-Laverde M (2002). Using niche-based GIS modeling to test geographic predictions of competitive exclusion and competitive release in South American pocket mice. Oikos.

[B47] Barton NH (1979). Gene flow past a cline. Heredity.

[B48] Zhang DX, Hewitt GM, Karp A, Isaac PG, Ingram DS (1998). Isolation of animal cellular total DNA. Molecular tools for screening biodiversity: plants and animals.

[B49] Zhang DX, Hewitt GM (1996). Nuclear integrations: Challenges for mitochondrial DNA markers. Trends Ecol Evo.

[B50] Yan LN, Zhang DX (2004). Effects of sample size on various genetic diversity measures in population genetic study with microsatellite DNA markers. Acta Zool Sin.

[B51] Park SDE (2001). Trypanotolerance in West African cattle and the population genetic effects of selection. PhD thesis.

[B52] Raymond M, Rousset F (1995). GENEPOP (v 1.2): Population genetics software for exact tests and ecumenicism. J Hered.

[B53] Lewis PO, Zaykin D (2001). GDA (Genetic Data Analysis): Computer Program for the Analysis of Allelic Data (version 1.0 d16c). http://lewis.eeb.uconn.edu/lewishome/software.html.

[B54] Excoffier L, Laval G, Schneider S (2005). Arlequin ver. 3.0: An integrated software package for population genetics data analysis. Evol Bioinfo Online.

[B55] Van Oosterhout C, Hutchinson WF, Wills DPM, Shipley P (2004). Micro-checker: software for identifying and correcting genotyping errors in microsatellite data. Mol Ecol Notes.

[B56] Goudet J (2001). FSTAT, a program to estimate and test gene diversities and fixation indices, version 2.9.3.

[B57] Felsenstein J (1993). PHYLIP (Phylogeny Inference Package) version 3.5c.

[B58] Takezaki N, Nei M (1996). Genetic distances and reconstruction of phylogenetic trees from microsatellite DNA. Genetics.

[B59] Chapuis MP, Estoup A (2007). Microsatellite null alleles and estimation of population differentiation. Mol Biol Evol.

[B60] Pritchard JK, Stephens M, Donnelly P (2000). Inference of population structure using multilocus genotype data. Genetics.

[B61] Rosenberg NA, Burke T, Elo K (2001). Empirical evaluation of genetic clustering methods using multilocus genotypes from 20 chicken breeds. Genetics.

[B62] Goudet J (1999). PCA-GEN for Windows, version 1.2.1.

