# Supplementary material for: Unexpected relationships of substructured populations in Chinese Locusta migratoria
Source: BMC Evol Biol. 2009 Jun 28;9:144. doi: 10.1186/1471-2148-9-144 (PMC2718889; doi:10.1186/1471-2148-9-144)
Supplement: Additional file 1 — Table S1. Pair-wise FST values of locust populations studies here (upper triangle: + statistically significant at 0.05 level; lower triangle: pair-wise FST; pop, population samples that are numbered as in Table 1; Neg, negative value, between -0.001 and -0.006) [file 1471-2148-9-144-S1.doc]

Pair-wise *F_ST_* values of locust populations studies here (upper triangle: + statistically significant at 0.05 level; lower triangle: pair-wise *F_ST_*; within-region comparisons are shown in bold print)

| pop | Ai | JLNA | BaM | Bo | HaM | HBAX | HBHH | HNLB | HNLK | HNMS | SDDY | SXYJ | sSXDL | TJ | AHYS | JSPX | LNHLD | LNJZ | hNLD | hNSYMS | HNSY1 | HNSY2 | L | SCJSJ | R | F |
| --- | --- | --- | --- | --- | --- | --- | --- | --- | --- | --- | --- | --- | --- | --- | --- | --- | --- | --- | --- | --- | --- | --- | --- | --- | --- | --- |
| Ai |  | **-** | **-** | **+** | **+** | - | - | - | - | - | - | - | - | - | + | - | - | - | **+** | **+** | **+** | **+** | + | + | + | + |
| JLNA | **0.008** |  | **-** | **+** | **+** | - | - | - | - | - | - | + | - | - | - | - | - | - | + | + | + | + | + | + | + | + |
| BaM | **0.000** | **0.008** |  | **+** | **+** | - | - | - | - | - | - | - | - | - | - | - | - | - | **+** | **+** | **+** | **+** | + | + | + | + |
| Bo | **0.037** | **0.024** | **0.035** |  | **+** | + | + | + | + | + | + | + | + | + | + | + | + | + | **+** | **+** | **+** | **+** | + | + | + | + |
| HaM | **0.015** | **0.015** | **0.011** | **0.046** |  | + | + | + | + | + | + | + | + | + | + | + | + | + | **+** | **+** | **+** | **+** | + | + | + | + |
| HBAX | 0.001 | 0.007 | 0.001 | 0.039 | 0.014 |  | **-** | **-** | **-** | **-** | **-** | **-** | **-** | **-** | **+** | **-** | **-** | **-** | **+** | **+** | **+** | **+** | + | + | + | + |
| HBHH | 0.003 | 0.003 | 0.002 | 0.031 | 0.011 | **0.001** |  | **-** | **+** | **-** | **-** | **+** | **-** | **-** | **+** | **-** | **-** | **+** | **+** | **+** | **+** | **+** | + | + | + | + |
| HNLB | 0.003 | 0.007 | 0.003 | 0.029 | 0.018 | **0.001** | **0.002** |  | **-** | **-** | **-** | **-** | **-** | **-** | **-** | **-** | **-** | **-** | **+** | **+** | **+** | **+** | + | + | + | + |
| HNLK | 0.005 | 0.007 | 0.003 | 0.035 | 0.015 | **0.003** | **0.004** | **0.000** |  | **-** | **+** | **-** | **-** | **-** | **+** | **-** | **-** | **-** | **+** | **+** | **+** | **+** | + | + | + | + |
| HNMS | -0.001 | 0.006 | 0.000 | 0.030 | 0.012 | **0.000** | **-0.002** | **0.000** | **-0.001** |  | **-** | **-** | **-** | **-** | **-** | **-** | **-** | **-** | **+** | **+** | **+** | **+** | + | + | + | + |
| SDDY | 0.004 | 0.004 | 0.001 | 0.035 | 0.012 | **0.002** | **0.002** | **0.001** | **0.004** | **-0.001** |  | **+** | **-** | **-** | **+** | **-** | **+** | **-** | + | + | + | + | + | + | + | + |
| SXYJ | 0.002 | 0.009 | 0.000 | 0.034 | 0.020 | **0.001** | **0.003** | **0.001** | **0.001** | **0.000** | **0.003** |  | **+** | **-** | **+** | **-** | **-** | **-** | + | + | + | + | + | + | + | + |
| sSXDL | 0.004 | 0.002 | 0.002 | 0.029 | 0.014 | **0.002** | **0.001** | **0.001** | **0.002** | **-0.002** | **0.001** | **0.003** |  | **-** | **+** | **-** | **-** | **-** | + | + | + | + | + | + | + | + |
| TJ | 0.000 | 0.004 | -0.003 | 0.031 | 0.013 | **-0.005** | **-0.003** | **-0.006** | **-0.004** | **-0.003** | **-0.004** | **-0.006** | **-0.003** |  | **-** | **-** | **-** | **-** | + | + | + | + | + | + | + | + |
| AHYS | 0.005 | 0.008 | 0.002 | 0.035 | 0.021 | **0.004** | **0.005** | **0.001** | **0.004** | **0.003** | **0.003** | **0.003** | **0.003** | **-0.003** |  | **-** | **-** | **-** | **+** | **+** | **+** | **+** | + | + | + | + |
| JSPX | 0.001 | 0.006 | 0.002 | 0.034 | 0.016 | **-0.001** | **0.000** | **0.002** | **0.001** | **0.000** | **0.002** | **0.002** | **0.000** | **-0.005** | **0.001** |  | **-** | **-** | + | + | + | + | + | + | + | + |
| LNHLD | 0.004 | 0.005 | 0.003 | 0.027 | 0.019 | **0.002** | **0.002** | **-0.002** | **0.000** | **0.001** | **0.004** | **0.002** | **0.002** | **-0.004** | **0.001** | **0.000** |  | **-** | + | + | + | + | + | + | + | + |
| LNJZ | 0.004 | 0.005 | 0.000 | 0.025 | 0.018 | **0.003** | **0.003** | **0.000** | **0.002** | **-0.001** | **0.003** | **0.002** | **0.001** | **-0.003** | **0.002** | **0.002** | **0.000** |  | + | + | + | + | + | + | + | + |
| hNLD | 0.036 | 0.032 | 0.040 | 0.060 | 0.059 | 0.041 | 0.038 | 0.030 | 0.031 | 0.033 | 0.037 | 0.035 | 0.033 | 0.027 | 0.040 | 0.034 | 0.033 | 0.036 |  | **-** | **-** | **-** | + | + | + | **+** |
| hNSYms | 0.056 | 0.048 | 0.061 | 0.073 | 0.084 | 0.061 | 0.057 | 0.046 | 0.047 | 0.056 | 0.053 | 0.051 | 0.048 | 0.049 | 0.055 | 0.054 | 0.048 | 0.049 | **0.007** |  | **-** | **-** | + | + | + | **+** |
| hNSY1 | 0.038 | 0.034 | 0.041 | 0.056 | 0.059 | 0.041 | 0.035 | 0.023 | 0.026 | 0.033 | 0.033 | 0.031 | 0.030 | 0.035 | 0.034 | 0.033 | 0.026 | 0.029 | **-0.004** | **-0.003** |  | **-** | + | + | + | **+** |
| hNSY2 | 0.053 | 0.039 | 0.057 | 0.067 | 0.073 | 0.058 | 0.053 | 0.044 | 0.043 | 0.049 | 0.050 | 0.051 | 0.046 | 0.043 | 0.054 | 0.051 | 0.044 | 0.049 | **0.002** | **0.004** | **-0.004** |  | + | + | + | **+** |
| L | 0.075 | 0.080 | 0.074 | 0.107 | 0.103 | 0.082 | 0.079 | 0.073 | 0.069 | 0.080 | 0.075 | 0.068 | 0.066 | 0.063 | 0.074 | 0.075 | 0.073 | 0.068 | 0.060 | 0.075 | 0.064 | 0.066 |  | **+** | **+** | + |
| SCJSJ | 0.117 | 0.108 | 0.120 | 0.117 | 0.149 | 0.128 | 0.117 | 0.109 | 0.107 | 0.119 | 0.116 | 0.106 | 0.104 | 0.110 | 0.112 | 0.113 | 0.107 | 0.103 | 0.077 | 0.089 | 0.085 | 0.071 | **0.119** |  | **+** | + |
| R | 0.093 | 0.086 | 0.089 | 0.118 | 0.124 | 0.098 | 0.095 | 0.086 | 0.084 | 0.093 | 0.087 | 0.085 | 0.078 | 0.080 | 0.088 | 0.089 | 0.087 | 0.080 | 0.068 | 0.070 | 0.069 | 0.063 | **0.025** | **0.112** |  | + |
| F | 0.071 | 0.062 | 0.074 | 0.082 | 0.103 | 0.081 | 0.077 | 0.063 | 0.064 | 0.072 | 0.070 | 0.066 | 0.062 | 0.061 | 0.070 | 0.070 | 0.067 | 0.063 | 0.029 | 0.034 | 0.028 | 0.027 | 0.059 | 0.074 | 0.053 |  |
